# Supplementary material for: Height-renderable morphable tactile display enabled by programmable modulation of local stiffness in photothermally active polymer
Source: Nat Commun. 2024 Mar 22;15:2554. doi: 10.1038/s41467-024-46709-7 (PMC10959967; doi:10.1038/s41467-024-46709-7)
Supplement: Supplementary file 1 — Supplementary Information [file 41467_2024_46709_MOESM1_ESM.pdf]

Supplementary Information

A height-renderable morphable tactile display enabled by programmable modulation of local stiffness in photothermally-active polymer

Inwook Hwang[1], Seongcheol Mun[1], Jung-Hwan Youn[1], Hyeong Jun Kim[2], Seung Koo Park[1], Meejeong Choi[1], Tae June Kang[2], Qibing Pei[3], Sungryul Yun[1,*]

[1]Tangible Interface Creative Research Section, Electronics and Telecommunications Research Institute, Daejeon, South Korea

[2]Department of Mechanical Engineering, Inha University, Incheon, South Korea

[3]Department of Materials Science and Engineering, Henry Samueli School of Engineering and Applied Science, University of California, Los Angeles, USA

*Corresponding Author. E-mail: sungryul@etri.re.kr

Supplementary Notes

Supplementary Note 1. Surface energy estimation via contact angle tests

Contact angle test for both P*t*BA and GNPE films was carried out using a KRÜSS DSA 25S drop shape analyzer. For the test, two probe liquids, a polar liquid (Deionized water) and a non-polar liquid (Diiodomethane), were individually dropped on each polymer surface. Contact angle of the liquid droplet formed onto the film surface is measured by a vision system of the equipment. Finally, we calculated surface energy of each film by exploiting Owens, Wendt, Rabel and Kaelble (OWRK) method (see equation (1-3)).

$$\frac{\sigma_L(1+\cos\theta)}{2} = (\sigma_S^D \cdot \sigma_L^D)^{1/2} + (\sigma_S^P \cdot \sigma_L^P)^{1/2} \quad (1),$$

$$\sigma_S^D = \frac{\sigma_L^D(1+\cos\theta)^2}{4},\ \sigma_S^P = \frac{\sigma_L^P(1+\cos\theta)^2}{4} \quad (2),$$

$$\sigma_S = \sigma_S^D + \sigma_S^P \quad (3)$$

Where σ_S is surface energy of solid (S), θ is contact angle with each liquid drop, and σ_S^D, σ_S^P, σ_L^D and σ_L^P are dispersive and polar components of surface energy for solid and liquid, respectively.

Supplementary Note 2. Estimation of the photothermal conversion efficiency of the GNPE

When light of wavelength λ is incident on a photothermal conversion material with power I_0, the absorbed light energy within the material is converted into thermal energy with a photothermal conversion efficiency, denoted as η_{PCE}. This converted heat flow causes a temperature increase in the material over time and some of this thermal energy is dissipated by convection to the atmosphere, expressed as $\dot{Q}_{loss}$, and to the substrate $\dot{Q}_{sub}$. The energy balance equation at thermal equilibrium can therefore be derived as follows [1-4]:

$$I_0\left(1-10^{-\alpha(\lambda)}\right) \times \eta_{PCE} = \sum mc_p \frac{dT}{dt} + \dot{Q}_{loss} + \dot{Q}_{sub}$$

where $\alpha(\lambda)$ is the absorbance of the material at a given wavelength λ, and mc_p is the thermal capacitance of the material and substrate.

The convective heat loss $\dot{Q}_{loss}$ is characterized as $hA(T-T_\infty)$, where h is the convective heat transfer coefficient, A is the surface area of the material for convection, T is a temperature of the material and T_∞ is the ambient temperature for the measurements. In a steady state, the temperature change with time ($\frac{dT}{dt}$) should be zero, and when a substrate with negligible photothermal conversion effect is used (*i.e.*, $\dot{Q}_{sub} = 0$), the photothermal conversion efficiency, η_{PCE}, can be simply expressed as follows:

$$\eta_{PCE} = \frac{hA(T-T_\infty)}{I_0(1-10^{-A(\lambda)})}$$

In this equation, the term hA is associated with the time constant (τ) of the specimen for the cooling process and is expressed as $\tau = \sum mc_p / hA$. This parameter can be determined experimentally by measuring the time taken for the temperature to fall from the steady-state temperature (T_{ss}) under light irradiation to $T_{ss} - 0.632(T_{ss} - T_\infty)$ during the cooling period.

To evaluate the photothermal conversion efficiency of GNPE, a 100 μm thick GNPE sample on PET substrate was cut into pieces, with an area of 36 mm × 33 mm. The GNPE sample was irradiated with a I_0 of 16.7 mW·cm^{-2} at a λ of 940 nm using an LED (Luminus SST 10-IRD-B50-U940) positioned at a distance of 62.5 mm above the sample. The light output of the LED was quantified using a photodetector (Newport 918D-ST-SL) connected to a handheld photometer (Newport 1919-R). Simultaneously, the temperature variation of the GNPE sample was recorded using a thermal camera (FLIR A6781 MWIR) at a frame rate of 50 frames per second, as shown in **Supplementary Fig. 10**. The material properties used to calculate the photothermal conversion efficiency are summarized in **Supplementary Table 1**. As a result, the photothermal conversion efficiency of GNPE is calculated to be 91.7%, which is comparable to those of carbon materials, which range from 83.73 to 93.4% [1-4].

Supplementary Note 3. Load-bearing test of the morphed shape

In our tactile display, each localized area is morphed into curved shape. Due to the geometrical feature, only for a single tactile cell, it is difficult to stably put a weight on the morphed area for the load-bearing test. Therefore, we first made out-of-plane deformation (deformation height: around 1250 μm) at four different areas by locally irradiating the NIR-LED light (light power: 1.1 W) under a pneumatic pressure of 18 kPa. The deformed areas, which were 12 mm distant from each other, formed protrusive vertices of a square. After generating the deformation, their deformed shape was latched by cooling down to ambient temperature under the pneumatic pressure and then reducing the pressure to atmospheric level. Finally, as shown in **Supplementary Fig. 11**, we carried out the load-bearing test by placing a glass substrate onto the deformed areas, putting the weight onto the substrate, and then measuring change in deformation height depending on the weight through a PSV-500 Laser scanning vibrometer.

Supplementary Note 4. Thermo-mechanical simulation of the photothermal-pneumatic actuation

a. Simulation conditions and the material properties

We carried out a numerical analysis using the finite element method to estimate the displacement profile and maximum displacement of P*t*BA-GNPE bilayer actuators as a function of temperature while maintaining a constant air pressure. The simulation software used was ANSYS Workbench 2021 R1 and the analysis included both steady-state thermal and static structural analyses. As the material properties of the bimorph layers exhibit a strong temperature dependence, we accounted for this non-linearity by enabling the Large Deflection option in the static structural analysis. The aim of this choice was to obtain more accurate results, taking into account the significant deformations experienced by the actuators.

To improve the convergence of the simulations, we used appropriate sub-stepping techniques. Specifically, in the Step Control panel, we initialized the number of sub-steps to 20, set the minimum sub-step to 10 and allowed a maximum of 100 sub-steps. This strategy ensured better convergence and stability during the iterative solving process. In addition, a multi-zone meshing technique was used to facilitate convergence in the numerical analysis. The mesh comprised a total of 363,432 nodes and 65,618 hexahedral elements, which optimized the representation of the physical system and allowed the accurate capture of complex deformations.

By implementing these approaches, we achieved a comprehensive analysis of the behavior of the P*t*BA-GNPE bilayer actuators under varying temperatures and constant pneumatic pressure, providing valuable insights into their mechanical response and performance. The

content of GNP in the GNPE was controlled at 0.5, 1, 2 and 5 wt%. The material properties of each sample, including Young's modulus, coefficient of thermal expansion and thermal conductivity, were determined experimentally and used for the simulations. The properties of P*t*BA and GNPEs as a function of temperature used in the simulation have been summarized and presented in **Supplementary Table 2**.

For the geometry, a bilayer of P*t*BA and GNPE, each 100-micron thick, was modelled as a disc with a diameter of 20 mm. The surface heat flux was then applied to the surface of a 4 mm circular region on the central surface of the GNPE. A fixed boundary condition was set on the bottom surface of the GNPE except for the area where the surface heat flux was applied. The initial temperature was set at 22 °C. Convective heat transfer was applied to the outer surface area with a convective heat transfer coefficient of 10.0 $W \cdot m^{-2} \cdot K^{-1}$. A pressure of 3 kPa was applied to the underside of the GNPE. The Ansys Parametric Design Language (APDL) code used to apply the Gaussian heat flux distribution and the material properties used for the simulation are given below:

[APDL code for the application of gaussian heat flux distribution]

```
c=arg1
a=1
b=1
d=4.714

*DIM,HEAT_FLX1,TABLE,6,19,1,,,,0
!
! Begin of equation: c*exp(-1*(({X}/a)^2+({Y}/b)^2)/d^2)
*SET,HEAT_FLX1(0,0,1), 0.0, -999
*SET,HEAT_FLX1(2,0,1), 0.0
*SET,HEAT_FLX1(3,0,1), c
*SET,HEAT_FLX1(4,0,1), a
*SET,HEAT_FLX1(5,0,1), b
*SET,HEAT_FLX1(6,0,1), d
*SET,HEAT_FLX1(0,1,1),        1.0,      -1, 0, 0, 0, 0, 0
*SET,HEAT_FLX1(0,2,1), 0.0, -2, 0, 1, 0, 0, -1
```

```
*SET,HEAT_FLX1(0,3,1),      0, -3, 0, 1, -1, 2, -2
*SET,HEAT_FLX1(0,4,1), 0.0, -1, 0, 1, 0, 0, -3
*SET,HEAT_FLX1(0,5,1), 0.0, -2, 0, 1, -3, 3, -1
*SET,HEAT_FLX1(0,6,1), 0.0, -1, 0, 1, 2, 4, 18
*SET,HEAT_FLX1(0,7,1), 0.0, -3, 0, 2, 0, 0, -1
*SET,HEAT_FLX1(0,8,1), 0.0, -4, 0, 1, -1, 17, -3
*SET,HEAT_FLX1(0,9,1), 0.0, -1, 0, 1, 3, 4, 19
*SET,HEAT_FLX1(0,10,1), 0.0, -3, 0, 2, 0, 0, -1
*SET,HEAT_FLX1(0,11,1), 0.0, -5, 0, 1, -1, 17, -3
*SET,HEAT_FLX1(0,12,1), 0.0, -1, 0, 1, -4, 1, -5
*SET,HEAT_FLX1(0,13,1), 0.0, -3, 0, 1, -2, 3, -1
*SET,HEAT_FLX1(0,14,1), 0.0, -1, 0, 2, 0, 0, 20
*SET,HEAT_FLX1(0,15,1), 0.0, -2, 0, 1, 20, 17, -1
*SET,HEAT_FLX1(0,16,1), 0.0, -1, 0, 1, -3, 4, -2
*SET,HEAT_FLX1(0,17,1), 0.0, -1, 7, 1, -1, 0, 0
*SET,HEAT_FLX1(0,18,1), 0.0, -2, 0, 1, 17, 3, -1
*SET,HEAT_FLX1(0,19,1), 0.0, 99, 0, 1, -2, 0, 0
! End of equation: c*exp(-1*(({X}/a)^2+({Y}/b)^2)/d^2)
!-->
sf,s1,hflux,%HEAT_FLX1%
```

b. Simulation results

Supplementary Fig.12a shows the deformation height of the P*t*BA-GNPE bilayer with different GNP contents in response to changes in temperature. As the temperature increases, the deformation rapidly increases and then gradually converges, as shown by the simulation results. It is also evident that the displacement with respect to temperature increases as the GNP content in the bilayer increases, which is mainly due to the decrease in Young's modulus of GNPE with increasing GNP content (as shown in Fig. 2h and 2i in the main text).

Supplementary Fig. 12b-d show the simulation results of the deformation height of the P*t*BA-GNPE bilayer with 2 wt% GNP content as a function of temperature. These simulations aimed to identify the influencing factors among the P*t*BA properties, namely coefficient of thermal expansion (CTE), thermal conductivity coefficient and Young's modulus, on the deformation of the bilayer. In **Supplementary Fig. 12b and c**, we compare the simulation

results with fixed values of CTE (147.5×10^{-6} K^{-1}) and thermal conductivity (146.627 $mW\cdot m^{-1}\cdot K^{-1}$) for P*t*BA. As shown in the figures, variations in the CTE and thermal conductivity of P*t*BA have negligible effects on the deformation height of the bilayer. **Supplementary Fig. 12d** shows a comparison between simulation results with a fixed value of elastic modulus for GNPE in P*t*BA (684.53 MPa) and simulation results taking into account the variation of elastic modulus with temperature. It is clear that the change in Young's modulus has a significant effect on the deformation of the bilayer.

These simulations provide valuable insights into the photothermal deformation behavior of P*t*BA-GNPE bilayers and lay the foundation for optimizing their operational performance. Furthermore, experimental evidence supports the feasibility of maximizing the height deformation of P*t*BA-GNPE bilayers by tailoring the properties of GNPE, in particular its elastic modulus. By systematically evaluating the effects of P*t*BA and GNPE properties, these results improve our understanding of bilayer behavior and aid in the design of high performance photothermal actuators.

Supplementary Figures

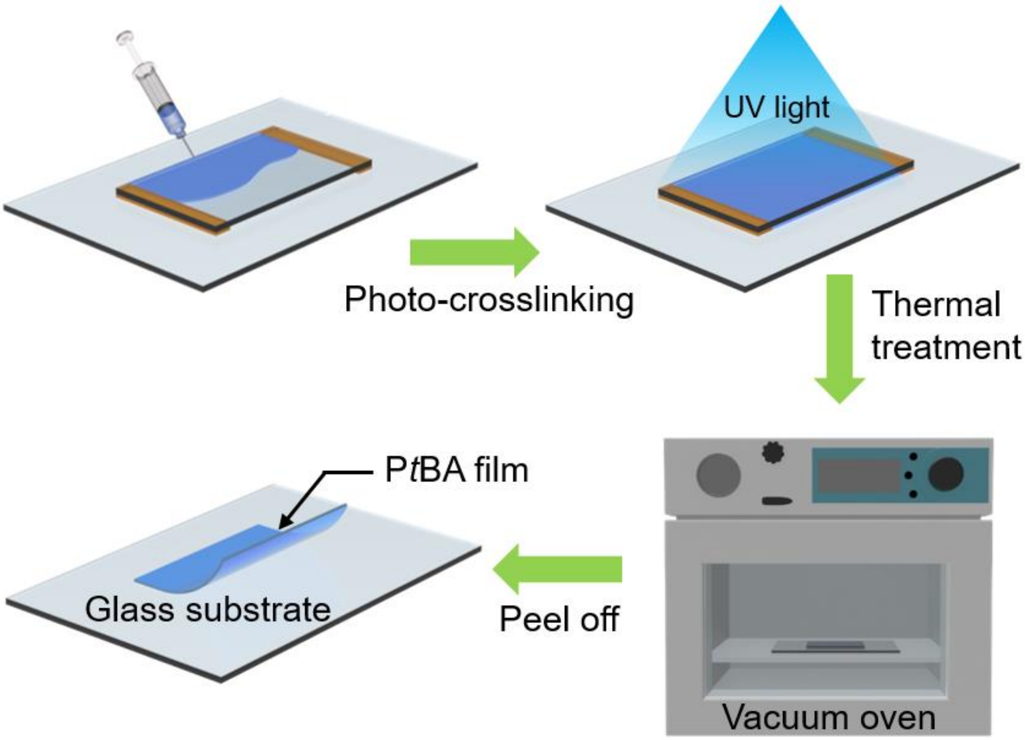

Supplementary Fig. 1. An illustrated fabrication process of the thin PtBA film

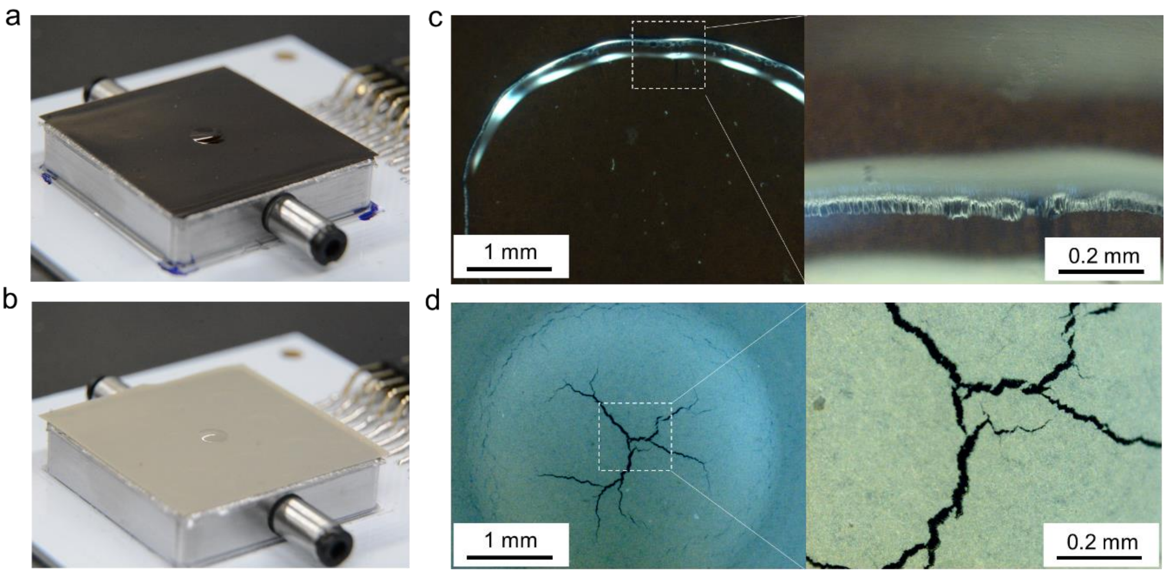

Supplementary Fig. 2. Photographs of the AgNWs and Tosylated PEDOT embedded PtBA films. a and b, Photothermal heating performance of tosylated PEDOT and AgNWs during

irradiation of NIR light. **c and d,** Microscopic observation of their surface after out-of-plane deformation under a pneumatic pressure of 6 kPa.

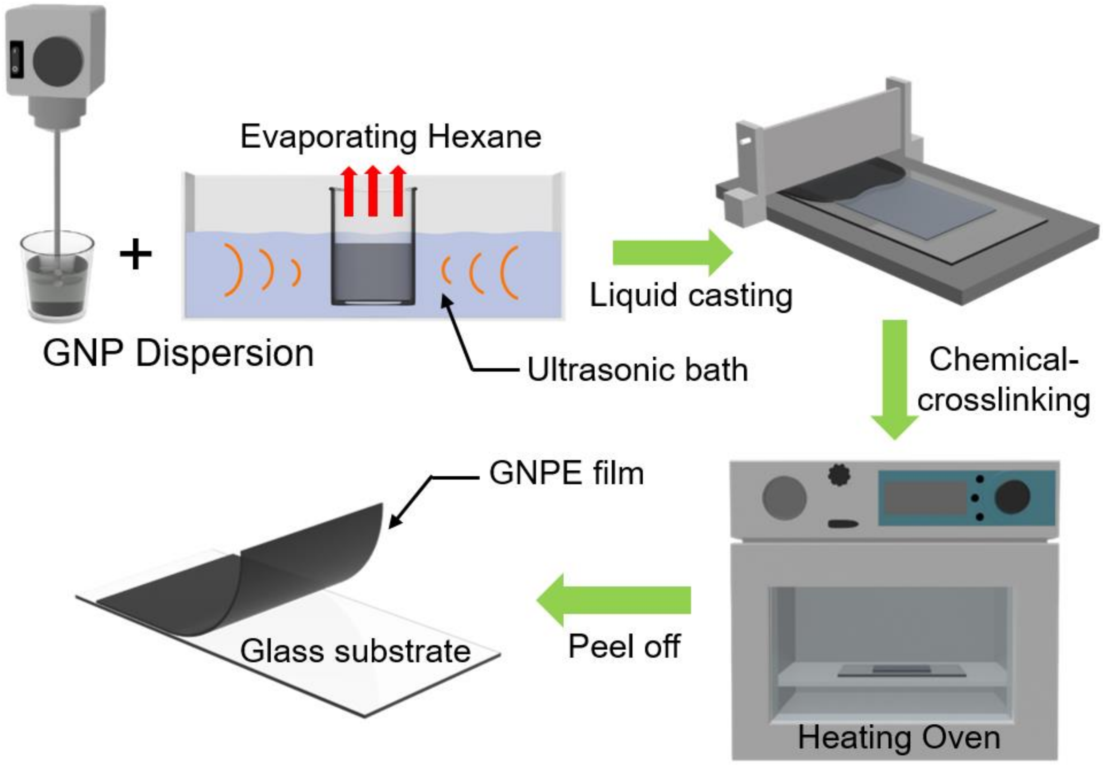

Supplementary Fig. 3. An illustrated fabrication process of the GNPE film

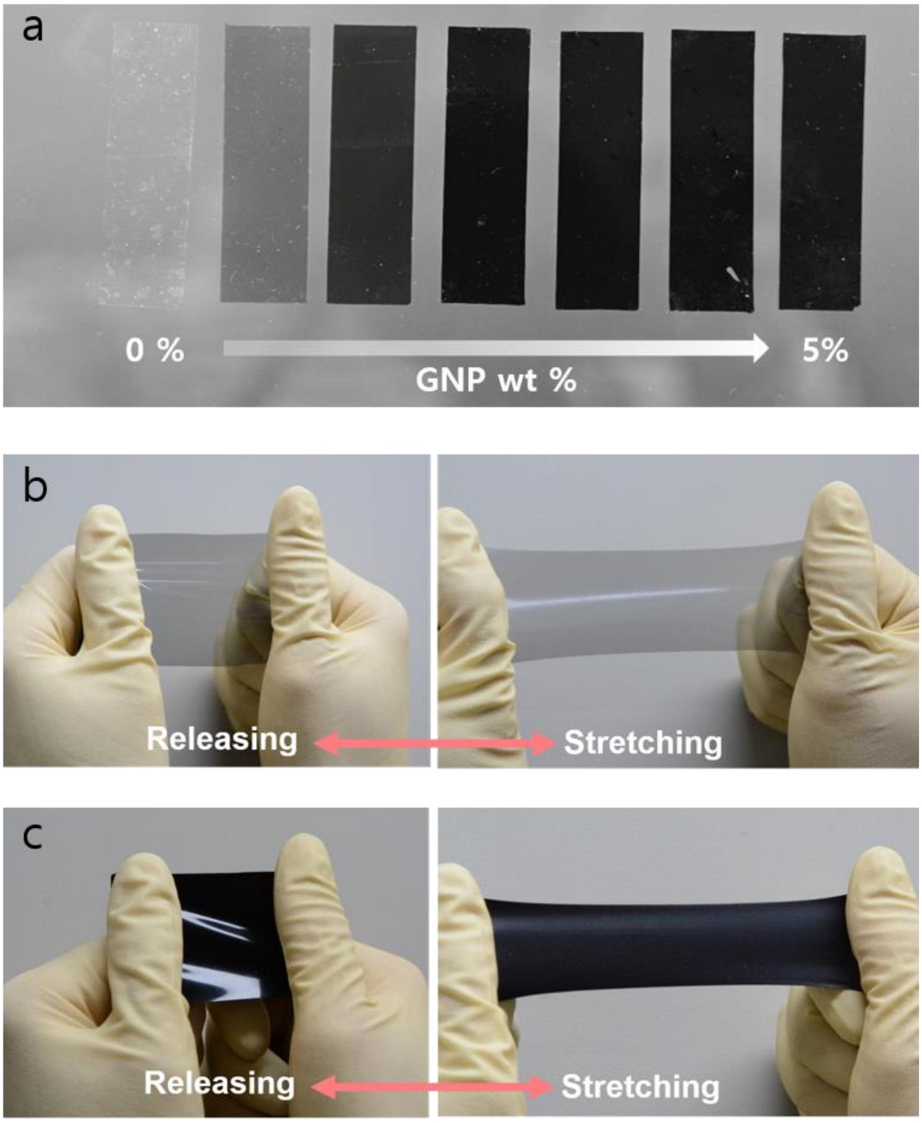

Supplementary Fig. 4. a, Photograph comparing transparency of the GNPEs with different GNP wt%. **b and c,** Photographs of elongation-recovery test of the GNPE membranes with different GNP wt%: b, GNPE 0.5 (GNP: 0.5 wt%) and c, GNPE 5.0 (GNP: 5.0 wt%).

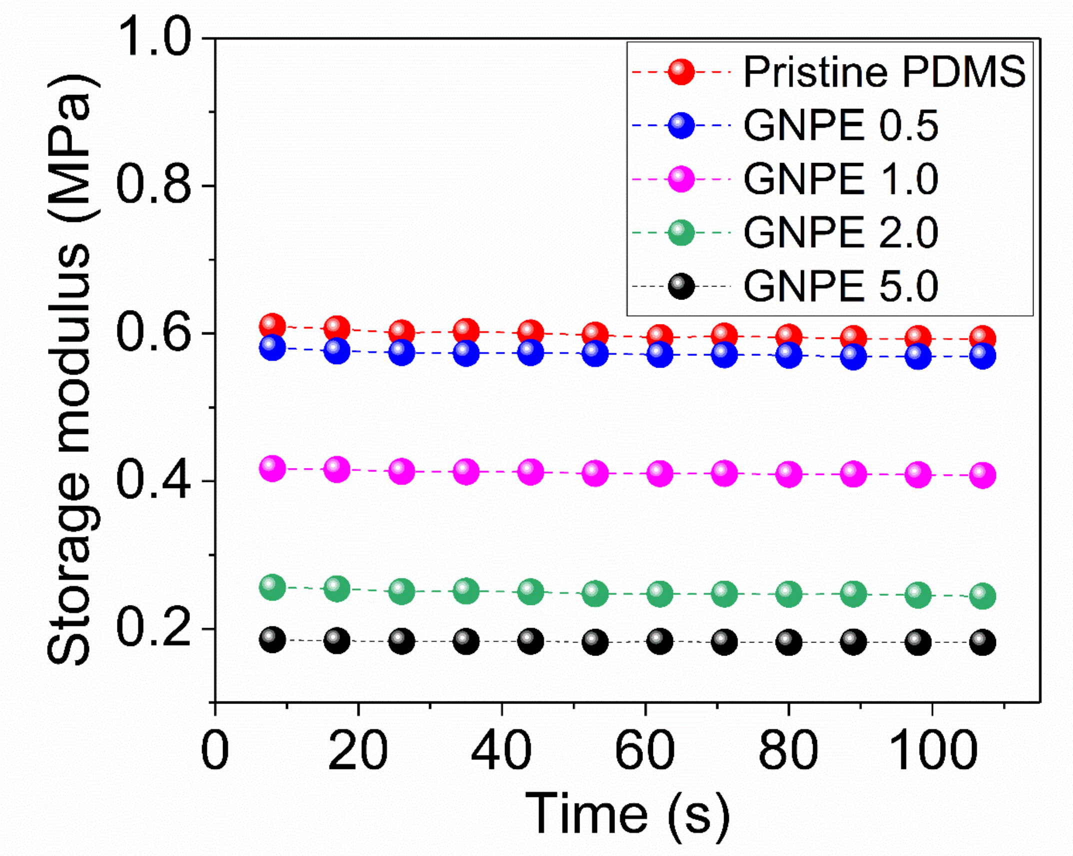

Supplementary Fig. 5. Storage modulus of the pristine PDMS and GNPEs at ambient conditions under a dynamic strain of 5%.

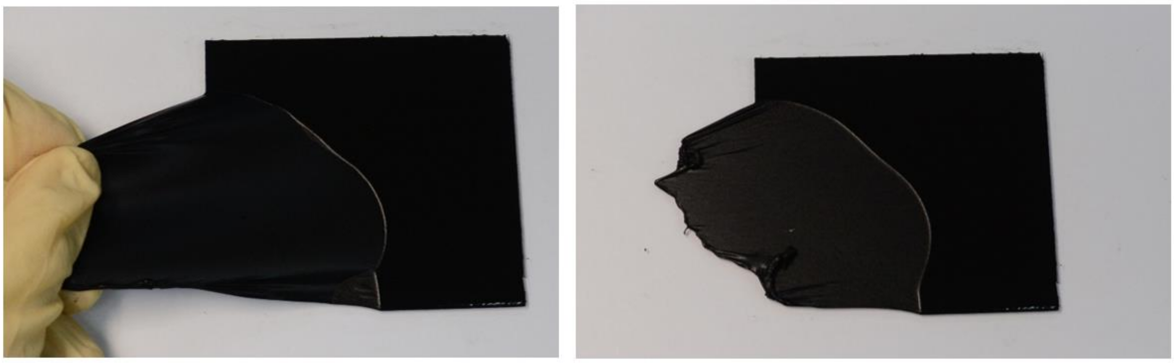

Supplementary Fig. 6. Photographs of elongation test of GNPE 10.0 film constructed on a glass plate after chemical crosslinking at 60 °C for 5 days in heating oven.

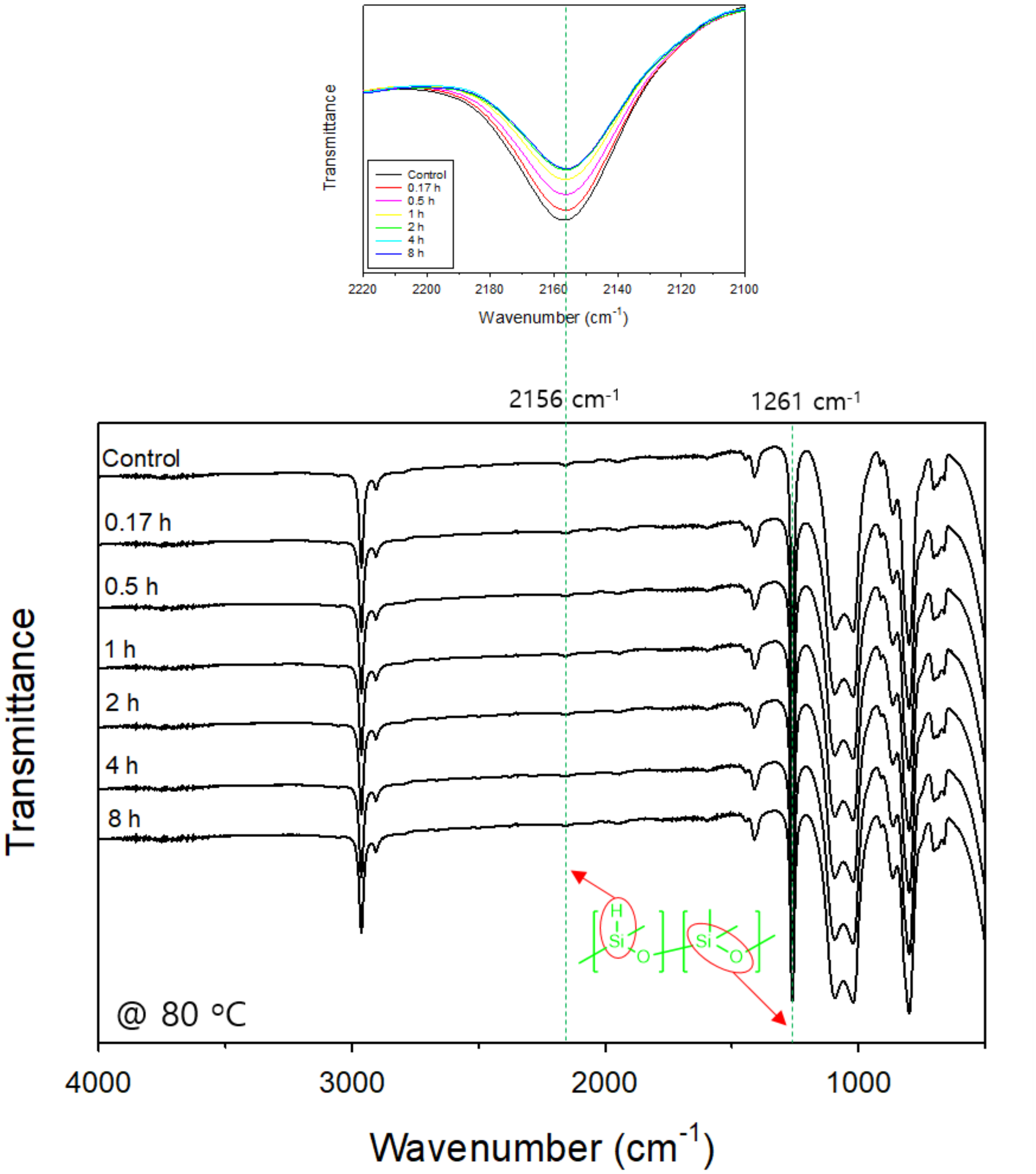

Supplementary Fig. 7. Reaction time dependent FT-IR spectra of the GNPE 5.0.

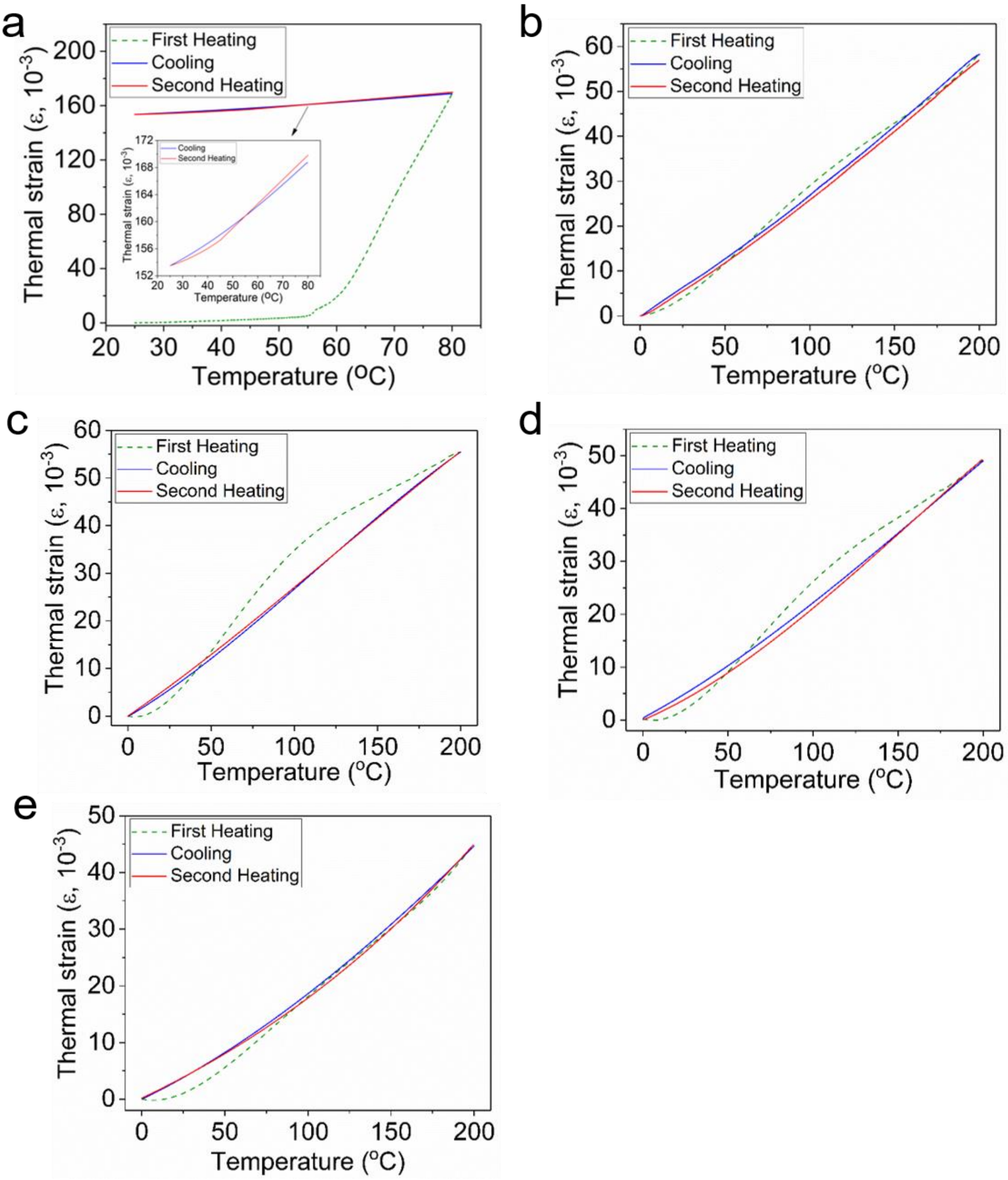

Supplementary Fig. 8. Temperature dependent thermal strain profile of the specimens under cyclic heating-cooling process. a, P*t*BA, **b-e,** GNPE 0.5, GNPE 1.0, GNPE 2.0, and GNPE 5.0, respectively.

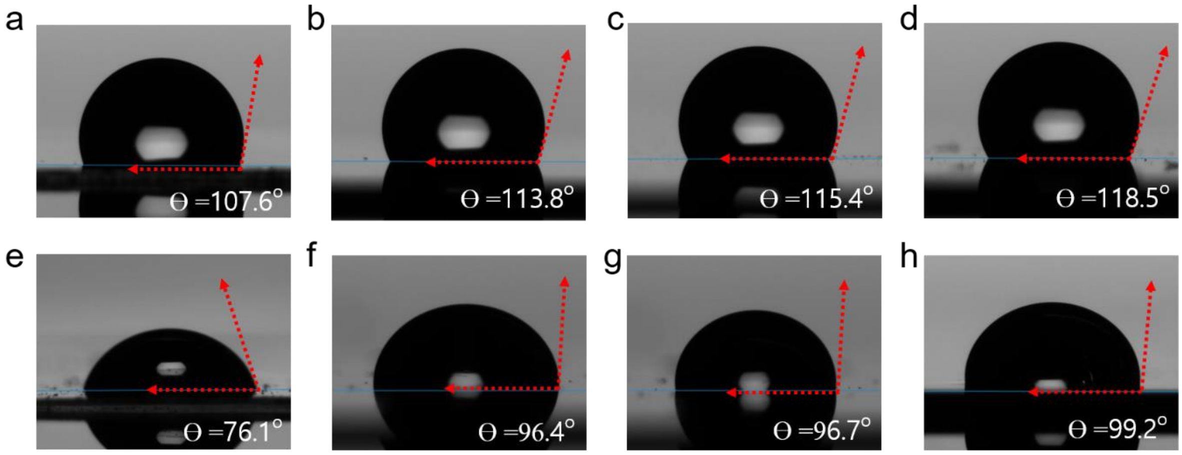

Fig. 9. Liquid contact angle measurement of the P*t*BA and the GNPE with different GNP contents. a-d, Contact angle of deionized water droplet (polar liquid) formed onto P*t*BA, GNPE 1.0, GNPE 2.0, and GNPE 5.0, respectively. **e-h,** Contact angle of diiodomethane droplet (non-polar liquid) formed onto P*t*BA, GNPE 1.0, GNPE 2.0, and GNPE 5.0, respectively.

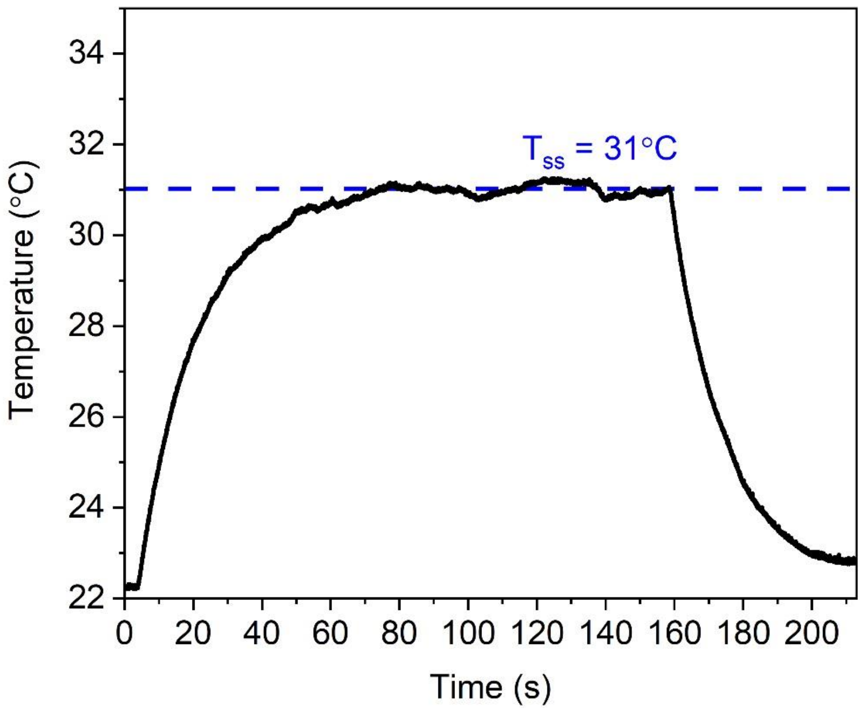

Supplementary Fig. 10. Temperature profile of GNPE measured under NIR-LED irradiation.

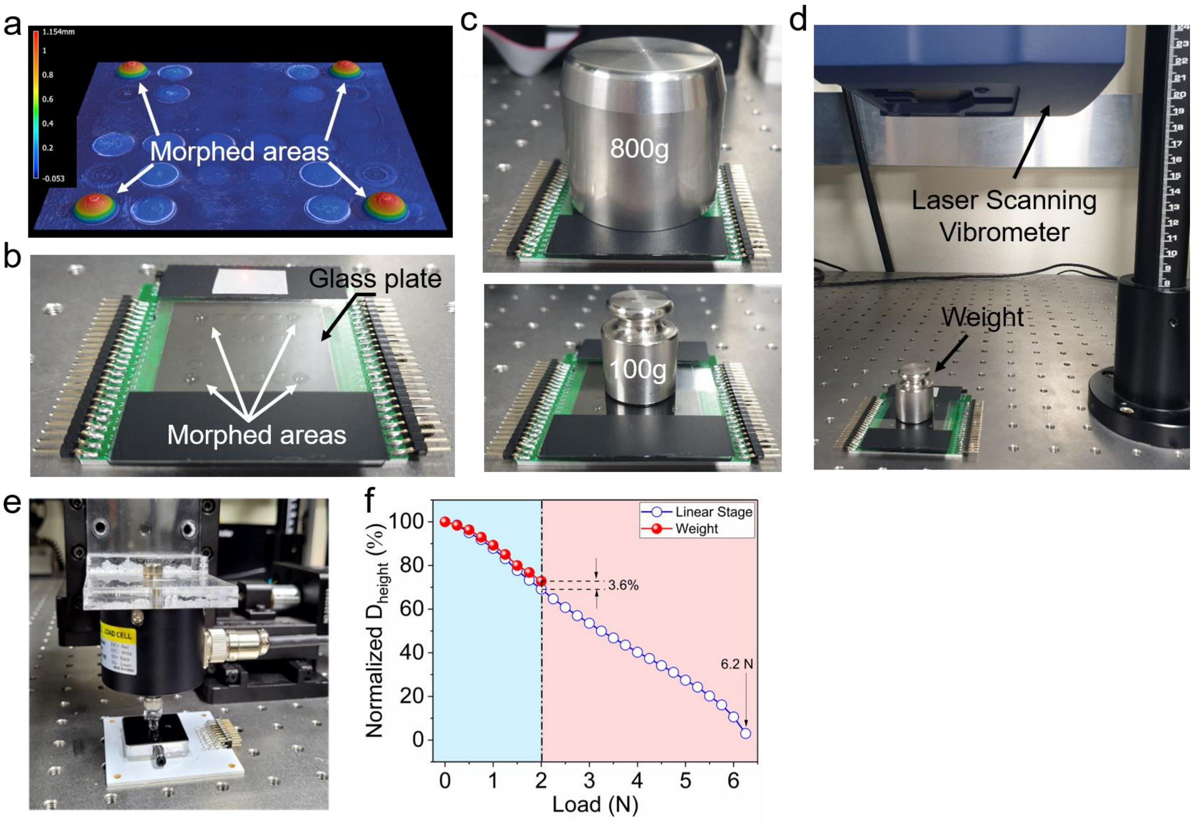

Supplementary Fig.11. Load-bearing test of the morphed areas after being latched. a, A 3D profiler image of tactile display with four morphed areas, equally spaced. **b and c,** Test set-up for applying load up to 2 N to each morphed area. **d,** Test environment using a laser scanning vibrometer for measuring change in deformation height with weight. **e,** Test environment using a load cell integrated into a linear stage. **f,** Comparison of load-D_{height} plots achieved from different methodology.

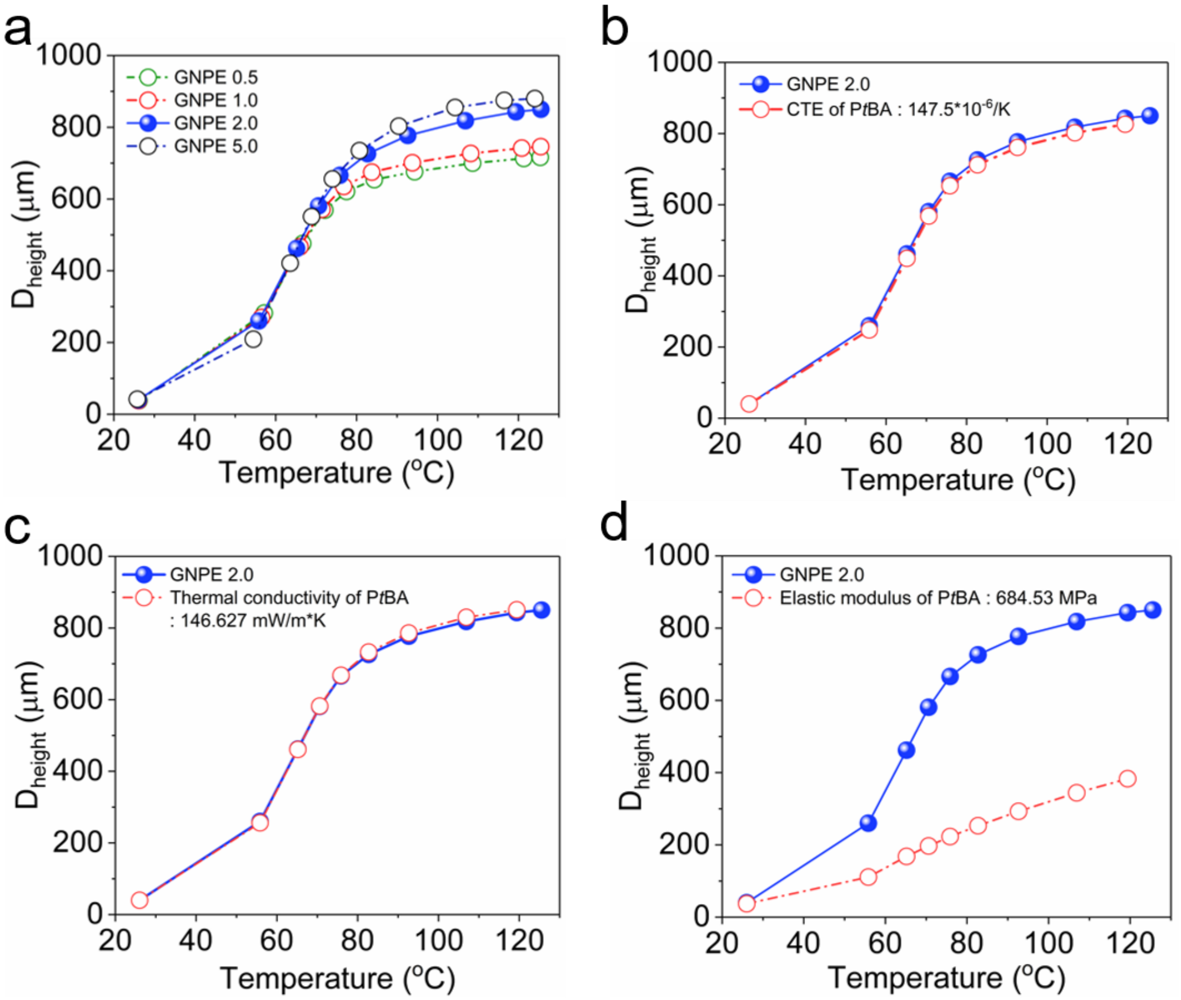

Supplementary Fig. 12. Thermo-mechanical simulation. a, The temperature dependent change in deformation height of P*t*BA-GNPE bilayer, adopting the GNPE layer with different GNP contents. **b-d,** Comparison of achievable deformation height between experimental data for P*t*BA-GNPE bilayer with GNPE 2.0 (colored in blue) and estimated results (red) adopting CTE of the P*t*BA as 147.5×10^{-6} K^{-1}, thermal conductivity of P*t*BA as 146.627 $mW{\cdot}m^{-1}{\cdot}K^{-1}$, and the elastic modulus of P*t*BA as 684.53 MPa, respectively.

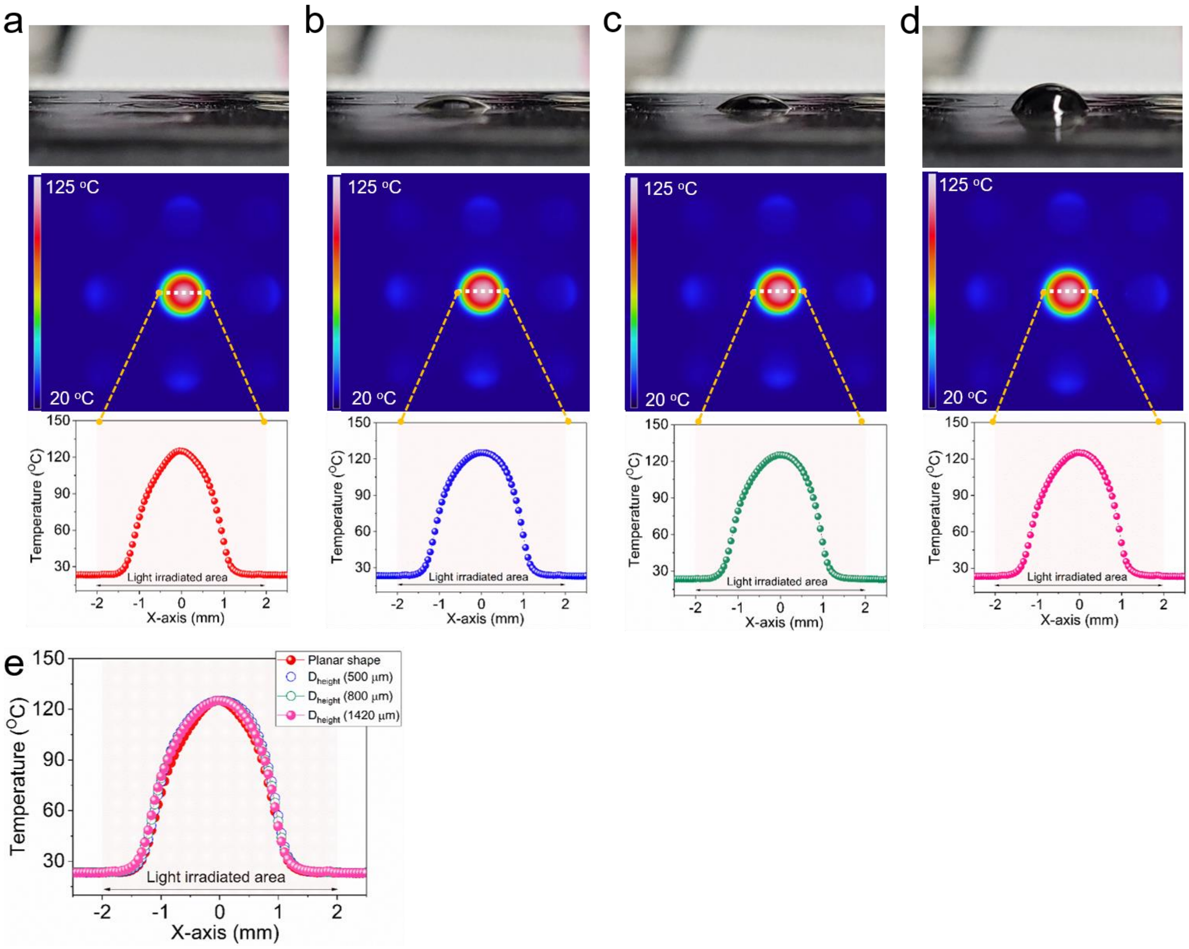

Supplementary Fig. 13. Comparison of temperature distribution on latched structure with different deformation heights during second photothermal heating through illumination of the LED light for three seconds with the same light power (1.4 W) to recover from the deformed state to the initial state. a, Planar shape, **b-d,** Deformed shape with deformation height of 0 μm, 500 μm, 800 μm, and 1420 μm, respectively. **e,** Comparison of temperature profiles achieved at target area.

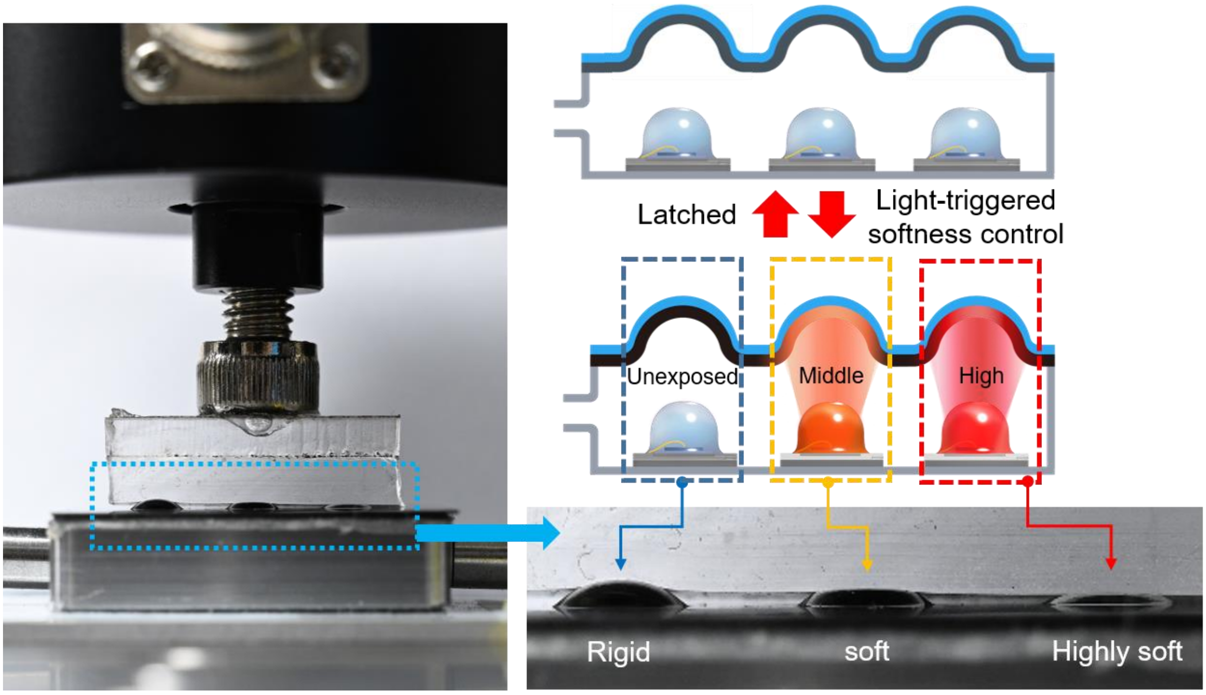

Supplementary Fig. 14. Softness control of individual morphed area. A photograph applying pressure to the morphed areas, which was latched, during localized irradiation of the light with different power. Since the morphed area is getting softer as temperature of the area increases, light power control of the individual LED for photo-thermal heating can change softness of the morphed areas to different levels. Therefore, as shown in the photograph, the deformation height of three morphed areas is reduced differently under a constant load.

Supplementary Tables

Supplementary Table 1. Parameters used to calculate the photothermal conversion efficiency of GNPE.

Parameter	GNPE	PET
Density, ρ [g·cm^{-3}]	1.054	1.37
Specific heat capacity, c_p [J·g^{-1}·K^{-1}]	1.415	1.0
Thermal capacitance, mc_p [mJ·K^{-1}]	33.556	30.825
Absorbance, $\alpha(940\ nm)$	1.4745	-

Steady-state temperature, T_{ss}: 31.03 ºC and ambient temperature, T_∞: 22.25 ºC
Time constant, τ: 16.4 s

Supplementary Table 2. Material properties used in the simulation.

Material property		Material				
		PtBA	GNPE0.5	GNPE1.0	GNPE2.0	GNPE5.0
Density ($g \cdot cm^{-3}$)		1.022	1.05	1.053	1.054	1.096
	Temperature (℃)					
Specific heat capacity ($J \cdot g^{-1} \cdot K^{-1}$)	25	1.418	1.486	1.424	1.415	1.293
	50	1.235	1.515	1.46	1.448	1.333
	100	1.95	1.563	1.531	1.514	1.411
	150	2.06	1.632	1.614	1.571	1.481
Coefficient of thermal expansion (10^{-6} K^{-1})	22-40	129.6				
	40-50	147.5				
	50-70		286	272	246	210
	70-120	328.9				
	100-150					
Thermal Conductivity ($mW \cdot m^{-1} \cdot K^{-1}$)	25	146.627	163.398	185.26	195.31	236.04
	50	139.7058	184.64	194.56	209.375	234.02
	100	261.52	213.14	211.91	224.735	254.41
	150	273.809	241.38	253.72	267.54	289.09
Young's modulus (MPa)	25	684.53	0.842	0.665	0.4522	0.333
	55	3.315	0.903	0.732	0.442	0.361
	65	0.925	0.908	0.714	0.47	0.378
	70	0.599	0.924	0.802	0.48	0.377
	75	0.4844	0.931	0.779	0.51	0.385
	85	0.3659	0.922	0.787	0.485	0.378
	95	0.2914	1.022	0.863	0.497	0.377
	110	0.212	1.033	0.854	0.526	0.491
	125	0.196	1.107	0.921	0.571	0.52

Supplementary Table 3. Performance comparison of tactile displays.

Driving force		Activated layer	Response time for deformation (s)	Change in D_{height} (Steps)	Maximum D_{height} (mm)	Maximum D_{height} /Diameter (mm/mm)	Reference
Joule heating	Pneumatic pressure	BS80-AA5	1	2	0.5-0.7	0.36-0.47	[5-7]
		MM4520	2.5	3	0.3-0.4	0.13	[8]
	Vapor pressure	Silicone elastomer	13	2	1.3	0.43	[9]
	SMA* (TiNiCu) actuation	Spring structure	Not provided	>14 (estimated)	0.08	0.053	[10]
Electrical Stimulus	Fluidic pressure	Silicone elastomer	Not provided	>14 (estimated)	0.05	0.1	[11]
			0.005	2	0.36-0.5	0.17	[12]
	Dielectric elastomer actuation		Not provided	14	0.471	0.24	[13]
	IPMC* actuation		1	>14 (estimated)	0.55	0.16	[14]
Photothermal heating	Volume phase transition	PNIPAAm	Several seconds	2	0.25	0.83	[15]
	Pneumatic pressure	P*t*BA-GNPE	3	14	1.42	0.36	Our work

*SMA and IPMC are short for shape memory alloy and ionic polymer-metal composite, respectively.

Supplementary References

1. Ren, H. *et al.* Hierarchical graphene foam for efficient omnidirectional solar–thermal energy conversion. *Adv. Mater.* **29**, 1702590 (2017).
2. Yang, H. *et al.* Developed carbon nanotubes/gutta percha nanocomposite films with high stretchability and photo-thermal conversion efficiency. *J. Mater. Res. Technol.* **9**, 8884-8895 (2020).
3. Luo, W. *et al.* Efficient enhancement of photothermal conversion of polymer-coated phase change materials based on reduced graphene oxide and polyethylene glycol. *J. Energy Storage* **78**, 109950 (2024).
4. Lv, S. *et al.* Flexible highly thermally conductive biphasic composite films for multifunctional solar/electro-thermal conversion energy storage and thermal management. *J. Clean. Prod.* **426**, 139004 (2023).
5. Qiu, Y., Lu, Z. & Pei, Q. Refreshable Tactile Display Based on a Bistable Electroactive Polymer and a Stretchable Serpentine Joule Heating Electrode. *ACS Appl. Mater. Interfaces* **10**, 24807-24815 (2018).
6. Xie, Z., Kim, J., Peng, Z., Qiu, Y. & Pei, Q. A 2D refreshable Braille display based on a stiffness variable polymer and pneumatic actuation. in *Electroactive Polymer Actuators and Devices (EAPAD) XXIII (eds. Madden, J. D., Anderson, I. A. & Shea, H. R.)* **21** (SPIE, 2021). doi:10.1117/12.2584039
7. Peng, Z. *et al.* Bistable electroactive polymers for refreshable tactile displays. in *Electroactive Polymer Actuators and Devices (EAPAD) XXI* (eds. Bar-Cohen, Y. & Anderson, I. A.) **67** (SPIE, 2019). doi:10.1117/12.2513780.
8. Besse, N., Rosset, S., Zarate, J. J. & Shea, H. Flexible Active Skin: Large Reconfigurable Arrays of Individually Addressed Shape Memory Polymer Actuators. *Adv. Mater. Technol.* **2**, 1700102 (2017).
9. Kwon, H.-J., Lee, S. W. & Lee, S. S. Braille dot display module with a PDMS membrane driven by a thermopneumatic actuator. *Sens. Actuator A Phys.***154**, 238–246 (2009).
10. Xu, J. *et al*. Fabrication and characterization of SMA film actuator array with bias spring for high-power MEMS tactile display. *Microelectronic Engineering* **227**, 111307 (2020).
11. Watanabe, J., Ishikawa, H., Arouette, X., Matsumoto, Y. & Miki, N. Artificial tactile feeling displayed by large displacement MEMS actuator arrays. in *2012 IEEE 25th International Conference on Micro Electro Mechanical Systems (MEMS)* 1129–1132 (IEEE, 2012).
12. Leroy, E., Hinchet, R. & Shea, H. Multimode Hydraulically Amplified Electrostatic Actuators for Wearable Haptics. *Adv. Mater.* **32,** 2002564 (2020).
13. Koo, I. M. *et al*. Development of Soft-Actuator-Based Wearable Tactile Display. *IEEE Trans. Robot.* **24**, 549–558 (2008).
14. Feng, G.-H. & Hou, S.-Y. Investigation of tactile bump array actuated with ionic polymer–metal

composite cantilever beams for refreshable braille display application. *Sensors and Actuators A: Physical* **275**, 137–147 (2018).

15. Richter, A. & Paschew, G. Optoelectrothermic Control of Highly Integrated Polymer-Based MEMS Applied in an Artificial Skin. *Adv. Mater.* **21**, 979–983 (2009).
