# Supplementary material for: Height-renderable morphable tactile display enabled by programmable modulation of local stiffness in photothermally active polymer
Source: Nat Commun. 2024 Mar 22;15:2554. doi: 10.1038/s41467-024-46709-7 (PMC10959967; doi:10.1038/s41467-024-46709-7)
Supplement: Supplementary file 3 — Description of Additional Supplementary Files [file 41467_2024_46709_MOESM3_ESM.pdf]

Description of Additional Supplementary Files

Supplementary Movie 1. A movie clip representing the character expression of our tactile display.

Supplementary Movie 2. A movie clip representing various shape expressions of our tactile display.

Supplementary Movie 3. A movie clip representing the texture expression with shape morphing of our tactile display.
